# Distributed Partial Discharge Locating and Detecting Scheme Based on Optical Fiber Rayleigh Backscattering Light Interference

**DOI:** 10.3390/s23041828

**Published:** 2023-02-06

**Authors:** Zhengxian Zhou, Hao Liu, Dawei Zhang, Yashuai Han, Xinyan Yang, Xianfeng Zheng, Jun Qu

**Affiliations:** 1 Anhui Province Key Laboratory of Optoelectric Materials Science and Technology, Anhui Normal University, Wuhu 241002, China; 2 School of Optical-Electrical and Computer Engineering, University of Shanghai for Science and Technology, Shanghai 200093, China

**Keywords:** optical fiber sensor, partial discharge, locating, backscattering light interference

## Abstract

Optical fiber sensors are used for partial discharge detection in many applications due their advantage of strong anti-electromagnetic interference capability. Multi-point distributed partial discharge detection and location are important for electrical equipment. In this paper, a distributed partial discharge location and detection scheme based on optical fiber Rayleigh backscattering light interference is experimentally demonstrated. At the same time, the location and extraction algorithm is used to demodulate the partial discharge signal; furthermore, the high-pass filter is used to reduce the system low-frequency noise and environment noise. It is clear that the proposed system can detect a partial discharge signal generated by metal needle sensitivity, and the detectable frequency range is 0–2.5 kHz. We carried out 10 locating tests for two sensing units, the experimental results show that the maximum location error is 1.0 m, and the maximum standard deviation is 0.3795. At same time, the signal-to-noise ratio (SNR) of sensing unit 1 and sensing unit 2 are greatly improved after demodulation, which are 39.7 and 38.8, respectively. This provides a new method for a multipoint-distributed optical fiber sensor used for detecting and locating a long-distance electrical equipment partial discharge signal.

## 1. Introduction

A healthy insulation state is a prerequisite for safe operation of electrical equipment [1]. Partial discharge is an early sign of electrical equipment’s insulation deterioration and a fatal factor that accelerates insulation damage. Therefore, effective detection and location of the partial discharge signals have important practical significance for electrical equipment [2]. Compared with traditional detecting methods, optical fiber sensors have many advantages such as high sensitivity, long sensing distance, strong corrosion resistance, inherent electrical insulation and immunity from electromagnetic interference, which have been widely used in the field of electric power to detect partial discharge signals [3,4,5].

At present, there are mainly three types of optical fiber partial discharge sensors: Fabry-Perot sensors (F-P) [6], fiber Bragg grating sensors (FBG) [7,8], and intrinsic interferometer sensors [9]. These types of sensors have different sensing properties in terms of sensitivity and frequency response due to their different structures, which have different applicability.

The fiber optic Fabry-Perot sensor has become a powerful tool for detecting weak partial discharge signals due to its inherent advantages. A Fabry-Perot sensor for partial discharge signal detection is demonstrated, of which a single-mode fiber and a graphene oxide is used as this sensing core [10]. Performance tests indicate that this proposed sensor maintains a linear acoustic-pressure response and a flat frequency response in the range of 200 Hz to 20 kHz, and the minimum PD size detected by this proposed sensor in air was approximately 100 pC. A suitable frequency bandwidth is important for fiber Fabry-Perot sensors in the detection of partial discharge signals. To improve frequency bandwidth performance for an F-P sensor, the frequency distribution of partial discharge signal is investigated, and an extrinsic F-P sensor is designed to detect partial discharge signals [11]. The results show that F-P sensors can effectively detect partial discharge emissions in both wideband and narrowband modes, and the intrinsic frequency of the F-P sensor should be designed in the frequency range of 100–170 kHz to obtain maximum sensitivity.

The fiber Bragg grating sensor is another important senor for partial discharge signal detection due to the high-frequency response, inherent electrical insulation and immunity from electromagnetic interference. A robust technique for classifying partial discharges in a controlled environment based on acoustic emissions captured using FBG sensors was demonstrated [12]. To improve the SNR of these signals, the use of the adaptive line enhancement-based technique is systematically explored through simulations, and the associated parameters are optimized. To overcome the low sensitivity of conventional methods, a partial discharge detection system based on phase-shifted fiber Bragg grating sensors was successfully developed [13]. Frequency response experiments indicate that the sensitivity of the phase-shifted fiber Bragg grating sensor is 8.46 dB higher than that of the conventional ultrasonic sensor.

In the past few years, fiber optic sensors based on an intrinsic interferometer have been applied to partial discharge detection [14,15]. An optical fiber Sagnac interferometer system is proposed for partial discharge ultrasound detection. Optical fiber sensing and time-frequency analysis of the ultrasonic signals excited by the piezoelectric ultrasonic transducer was realized for the first time [16]. Experiments showed that with a voltage of 10 kV, the time-domain amplitude range of the partial discharge signals was 0.8~1.9 V, and the frequency response range was up to 58 kHz. The Michelson sensor is another intrinsic interferometer sensor for partial discharge detection. A simulation model of the response sensitivity of the Michelson partial discharge sensing system was proposed, and the average response sensitivity of the proposed sensing system is higher than that of the conventional PZT system [17].

The F-P, FBG, Sagnac and Michelson sensors have been widely investigated regarding their use in partial discharge sensing. However, these sensors are mostly used in places with limited space and can only measure a single point, which is not suitable for long-distance distributed partial discharge detection and location.

In this paper, a distributed partial discharge sensing scheme based on optical fiber Rayleigh backscattering light interference is experimentally demonstrated. This system can locate and detect partial discharge signals generated by metal needle sensitivity. This provides a new method for multipoint-distributed optical fiber sensor used for detecting and locating a long-distance electrical equipment partial discharge signal.

## 2. Theory of Optical Fiber Rayleigh Backscattering Light Interference

**Figure 1:**
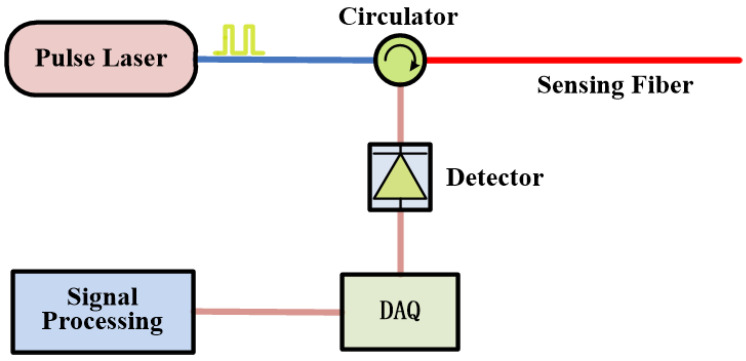
System configuration of OTDR.

The optical time-domain reflectometry (OTDR) has been widely used in optical fiber communication and sensing systems to locate backscattering signals that come back from optical fibers [18,19]. The system diagram is shown in Figure 1.

**Figure 2:**
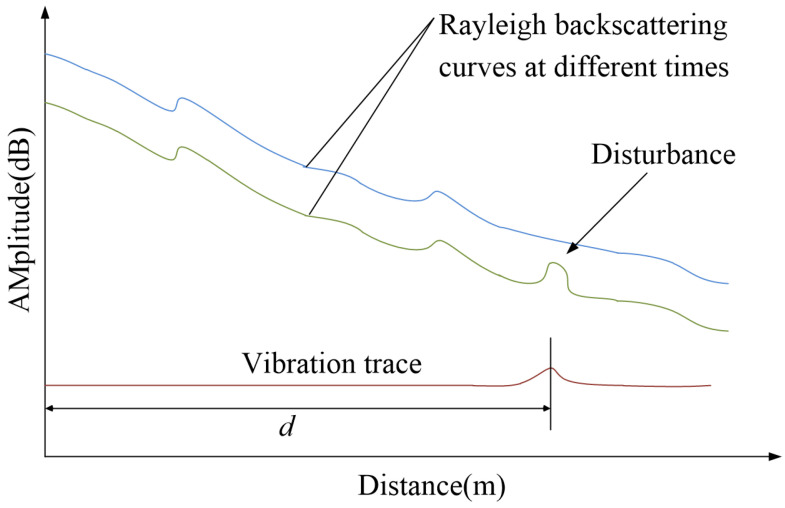
Locating schematic diagram of disturbance signal based on OTDR.

As shown in Figure 1, the pulse light emitted by the laser is injected into the sensing fiber through the optical circulator, which generates backscattering light in the fiber for disturbance sensing. The backscattering signals are digitized by a data acquisition card (DAQ) after being converted into analog electrical signals by a photodetector, and then, they are saved to a solid-state disk for further processing. When there is a disturbance in the optical fiber, the refractive index of the fiber changes, which will change the intensity of the backscattered light at the corresponding location. The location of the disturbance signal can be calculated by the principle of optical time-domain reflectometry, as shown in Equation (1).

(1) $$d=\frac{c\;\cdot \;\Delta t}{2n}$$

where Δt is the time it takes for an optical signal to be emitted and returned, c is the speed of light in a vacuum, and n is the refractive index of the fiber. From Equation (1), we see that the location of the disturbance signal can be calculated by Δt, as shown in Figure 2.

**Figure 3:**
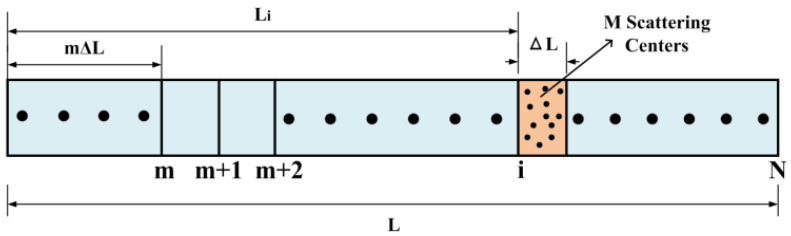
Rayleigh backscattering light interference model.

The partial discharge signal is a kind of disturbance signal that can be detected by Rayleigh backscattering light interference in a sensing fiber. The pulse light propagating forward in the optical fiber produces Rayleigh backscattering light, in which interference effects will occur within one pulse period. The interference effects can be simulated by a simple discrete model [20,21], as shown in Figure 3.

Figure 3 shows the discrete model of Rayleigh backscattering light interference. There are N small backscattering zones on the optical fiber of length L, in which each zone has length ΔL, ΔL = L/N. The Rayleigh backscattered light in the whole sensing fiber can be represented by N scattering zones. The backscattering light intensity in i-th scattering zones is the coherent addition of randomly distributed scatters within the region ΔL, and the number of scattering centers in each zone is M. Therefore, the Rayleigh backscattering light interference field at distance L_i_ can be expressed by

(2) $$\vec{E_{b}}\left(L_{i}\right)=E_{0}e^{-\alpha \left(i-1\right)∆L}\sum_{k=1}^{M}\alpha_{k}^{i}e^{{j\varphi }_{k}^{i}}$$

where E_0_ is the electric field intensity of incident light, α is the attenuation coefficient of the optical sensing fiber, and $\alpha_{k}^{i}$ and $\varphi_{k}^{i}$ are the amplitude and phase of *k*-th scatter center in *i*-th scattering zone, respectively. As shown in (2), the interference intensity between Rayleigh backscattering light in an optical sensing fiber can be expressed as a vector summation of the amplitude and phase of the scattering centers within one region.

## 3. System Configuration and Signal Processing

### 3.1. System Configuration

**Figure 4:**
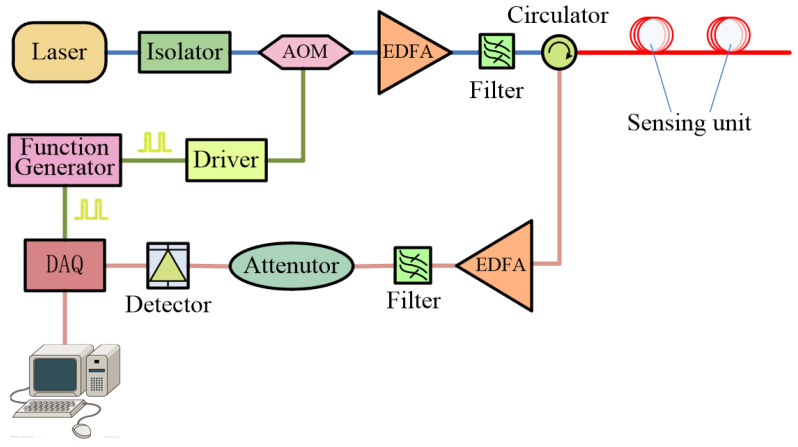
System configuration of distributed partial discharge detection.

In this paper, a distributed partial discharge detection system based on optical fiber Rayleigh backscattering light interference is experimentally demonstrated, in which the Rayleigh backscattering light is used to measure the vibration signals generated by electrical equipment discharge. The system configuration is shown in Figure 4.

As shown in Figure 4, the laser with a central wavelength of 1550.12 nm and 1 kHz linewidth emits continuous light waves that are chopped into pulses by an acoustic optical modulator (AOM, Gooch & Housego; wavelength: 1530–1560 nm; frequency shift: 200 MHz).The pulses are amplified to 30 dB by an erbium-doped fiber amplifier (EDFA) and then injected into the sensing fiber (Corning SMF-28e+) through the optical circulator after being filtered. The backscattering light from the sensing fiber is amplified by another EDFA and then converted into analog electrical signals by a detector after being filtered. Following, the Rayleigh backscattering signals are digitized by a data acquisition card (DAQ, Spectrum M4i.4450-x8) and are saved to a solid-state disk (SSD) for further processing. The function generator generates pulse signals to drive AOM through the radio frequency driver and, at the same time, outputs synchronous pulse signals to DAQ for data synchronization sampling. The interference intensity of Rayleigh backscattered light will change when there is partial discharge disturbance around the fiber, which will be extracted through a signal-processing unit in the end.

### 3.2. Signal Processing

**Figure 5:**
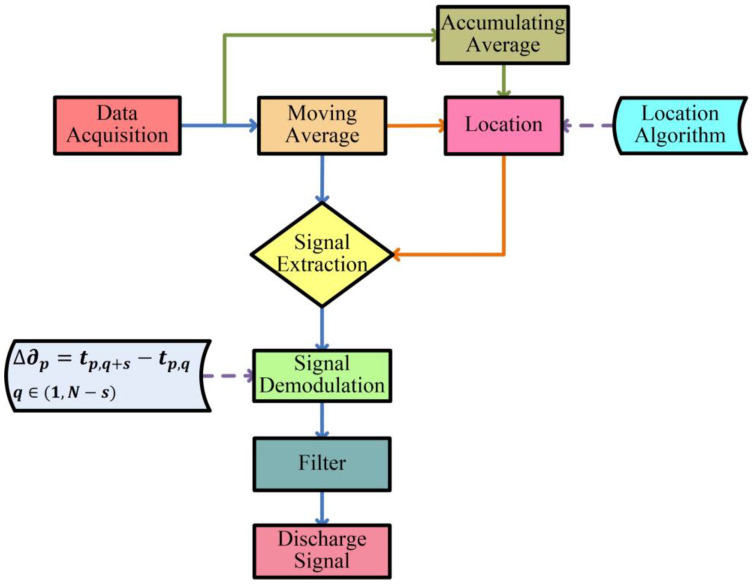
Signal processing diagram.

The partial discharge location and detection scheme includes data acquisition and preprocessing, signal locating, partial discharge extraction and demodulation. The location and detection scheme diagram is shown in Figure 5.

As shown in Figure 5, the original data are collected by a data acquisition card, and then, the moving average is performed for preprocessing. At the same time, the location information is obtained by cumulating the average and location algorithm. In the end, partial discharge signals are extracted and demodulated according to location information.

#### 3.2.1. Signal Acquisition and Preprocessing

First, 51,200 original Rayleigh backscattering curves were collected, in which each curve include 27,000 data points. According to OTDR theory, a 500 MHz sampling rate corresponds to a 0.2-m spatial interval [22,23,24]. The collected data are expressed as a matrix:

(3) $$t_{i,j}=\left\{t_{i,1},t_{i,2},\ldots ,t_{i,j},\ldots ,t_{M,N}\right\}$$

where M = 27,000, N = 51,200, i∈(1,M), j∈(1,N).

Due to the inherent defects in the sensing system, the original Rayleigh backscattered signal contains thermal noise, shot noise, and electrical noise. These noises submerge part of the partial discharge signal and distort the extracted signal. In order to reduce these random noises, it is necessary to process all the curves of the collected data with a moving average, as follows:

(4) $$A_{j}=\frac{1}{L}\sum_{l=i}^{i+L-1}t_{l,j}$$

where L is the step length of the moving average, l is the length of each step, and l∈(i,i+L−1). A new matrix is obtained from the original data t processed by the moving average:

(5) $$A_{j}=\left\{A_{m,1},A_{m,2},\ldots \ldots \ldots A_{m,j}\ldots \ldots ,A_{m,N}\right\}$$

where m∈(1,M−L+1).

#### 3.2.2. Signal Location

Since the average random noise of the system and environment is close to zero, the cumulative averaging is used to reduce the fluctuation of the longitudinal amplitude of the signal. After the cumulative average is calculated for all the curves of the original data t, a new curve is obtained as follows:

(6) $$B=\frac{1}{N}\sum_{j=1}^{N}t_{i,j}$$

After that, all the original curves t minus B to obtain the difference value Δt, as follows:

(7) $$∆t_{i,j}=t_{i,j}-B$$

(8) $$∆t=\left\{∆t_{i,1},∆t_{i,2}\ldots ∆t_{i,j}\ldots ∆t_{i,N}\right\}$$

Then, n curves from ∆t are taken to calculate the time-domain variance, in which it moves n curves each time after finishing the current step, as follows:

(9) $$E_{i,k}=\left\{\frac{1}{n}\cdot \sum_{w=n\cdot k-n+1}^{n\cdot k}{\left(∆t_{i,w}-P_{i,\mathrm{ave}}\right)}^{2}\right\}$$

$$P_{i,\mathrm{ave}}=\frac{1}{n}\overset{n\cdot k}{\underset{w=n\cdot k-n+1}{\sum }}∆t_{i,w}$$

where w∈(n·k−n+1,n·k), k=N/n.

Then, to obtain the k variance curves:

(10) $$E_{i,v}=\left\{E_{i,1},E_{i,2}\ldots E_{i,v}\ldots E_{i,k}\right\}$$

The k variance curves are superimposed and averaged to obtain the final variance average curve $E_{i,\mathrm{ave}}$, as follows:

(11) $$E_{i,\mathrm{ave}}=\frac{1}{k}\sum_{v=1}^{k}E_{i,v}$$

From Equation (11), the peak position of the curve is obtained, which is the position of the partial discharge signal on the sensing optical fiber.

#### 3.2.3. Signal Extraction and Demodulation

The partial discharge signal at peak position is extracted from the original Rayleigh backscattering curves $t_{i,j}$ according to location information which is obtained from Equation (11), as follows:

(12) $$t_{p}=\left\{t_{p,1},t_{p,2},\ldots \ldots \ldots ,t_{p,N}\right\}$$

where p is the peak position of the partial discharge signal on the fiber. The moving subtraction is used to demodulate the partial discharge disturbance signal, as follows:

(13) $$∆\partial_{p}=t_{p,q+s}-t_{p,q}$$

where s is the step of moving subtraction, q∈(1,N−s). Then, a difference curve with N-s data points is obtained by Equation (13), as follows:

(14) $$∆\partial_{p}=\left\{∆\partial_{1},∆\partial_{2},\ldots \ldots \ldots ,∆\partial_{N-s}\right\}$$

In the end, the demodulated partial discharge signal is filtered by a high-pass filter to reduce the system low-frequency noise and environment noise according to its spectrum range.

## 4. Experiments and Results

### 4.1. Experimental Setup and Parameters

**Figure 6:**
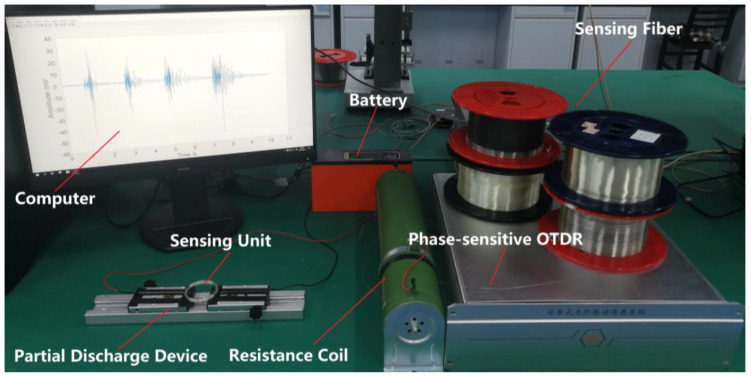
Experimental setup of partial discharge sensing system.

The experimental setup is shown in Figure 6, in which two partial discharge sensing units are set at 1560 and 2135 m of sensing fibers, respectively.

The partial discharge test system consists of a partial discharge device, a 48 V lithium battery, a resistance coil, a phase-sensitive OTDR, a 5400-m sensing fiber and two sensing units. In the experiment, the laser with 1 kHz line width generates a 10 mW continuous light wave that is then modulated into a sequence of pulses with a 5 kHz repetition rate and 50 ns pulse width by AOM. A 5400-m Corning single-mode fiber is used as the optical sensing fiber, and two sensing units with 5 cm diameter and 5 m length are set 1560 and 2135 m away from the beginning of sensing fiber. The partial discharge device is powered by a 48 V lithium battery to generate the partial discharge signal, and a resistance coil is used in the circuit to limit the discharge current. Then, the partial discharge signal is detected by two sensing units synchronously. At the same time, a high-speed data acquisition card with 500 MS/s sampling rate is used to collect the Rayleigh backscattering signals, which return from the sensing optical fiber.

### 4.2. Experimental Results and Discussion

**Figure 7:**
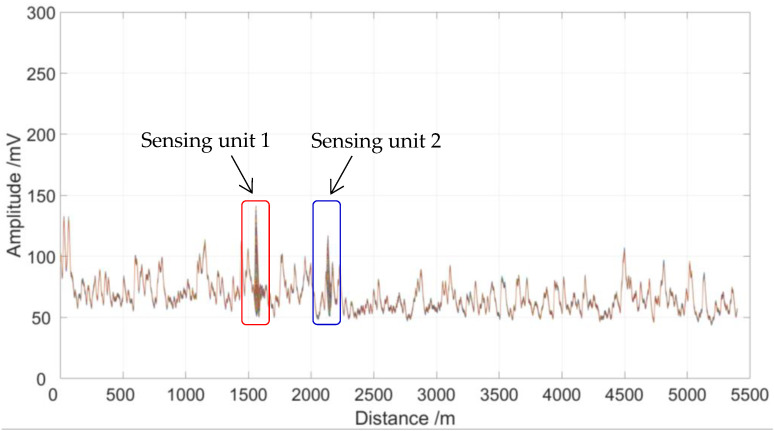
Rayleigh backscattering signals with partial discharge disturbance.

During the experiment, two sensing fiber coils with 5 cm diameter and 5 m length are set 1560 and 2135 m away from the beginning of sensing optical fiber. The partial discharge metal electrode is located at the center of the two sensing fiber coils, which is powered by a 48 V lithium battery to generate a partial discharge signal, and a resistance coil is used in the circuit to limit the discharge current. Firstly, 50,000 original Rayleigh backscattering signals with a length of 5400 m from the sensing fiber were collected by a 500 MS/s data acquisition card, which was then processed by using the moving average to improve the SNR. Finally, the Rayleigh backscattering signals with partial discharge disturbance were obtained, as shown in Figure 7.

Based on sampling and OTDR principles, it takes 10 s to collect 50,000 original Rayleigh backscattering signals at a 5 kHz pulse repetition rate. A 500 MS/s sampling rate corresponds to a 0.2-m space interval on the optical fiber; that is, there are 27,000 data points in one original Rayleigh backscattering trace.

As shown in Figure 7, there are two partial discharge disturbance signals after 1500 and 2000 m, but the location information is vague, which is affected by the spatial resolution and data sampling rate of the distributed optical fiber sensing system. Pulse light width and data sampling rate are the two main factors affecting the spatial resolution of the distributed optical fiber sensing system. The 500 MS/s sampling rate corresponds to the 0.2-m space interval on the optical fiber, which limits the locating accuracy of the system to no better than 0.2 m. On the other hand, according to the theory of OTDR, spatial resolution Δd can be expressed as

(15) $$\Delta d=\frac{c\cdot W}{2n}$$

where W is the pulse light width, c is the speed of light in a vacuum, and n is the refractive index of the fiber. From Equation (15), we see that a 50 ns pulse light width corresponds to 5 m of spatial resolution. According to the sampling rate and spatial resolution, we see that the locating accuracy of the distributed optical fiber system is no better than 5 m.

**Figure 8:**
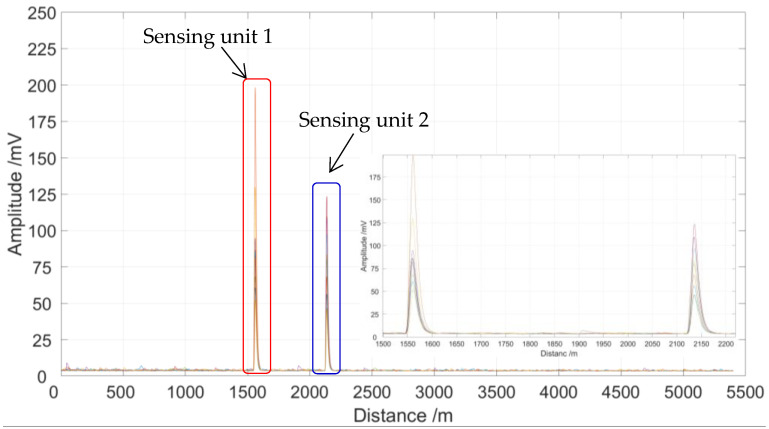
Ten partial discharge location curves.

In order to improve the system locating performance, a locating algorithm based on time-domain variance is proposed and tested. The partial discharge location curves after 10 locating tests, processed by the time-domain variance locating algorithm, are shown in Figure 8.

**Figure 9:**
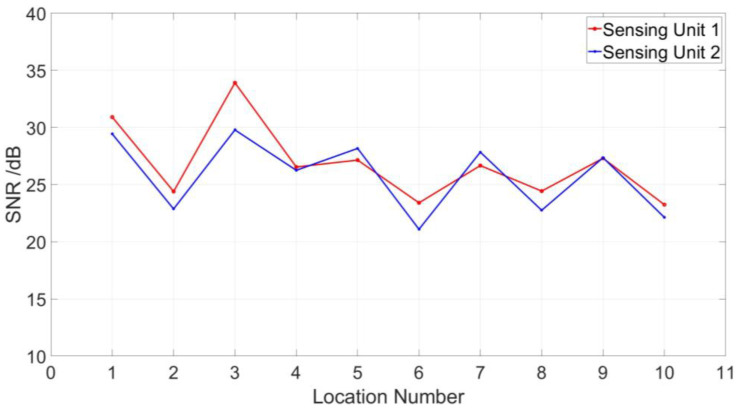
Signal-to-noise ratio of 10 partial discharge location curves.

**Figure 10:**
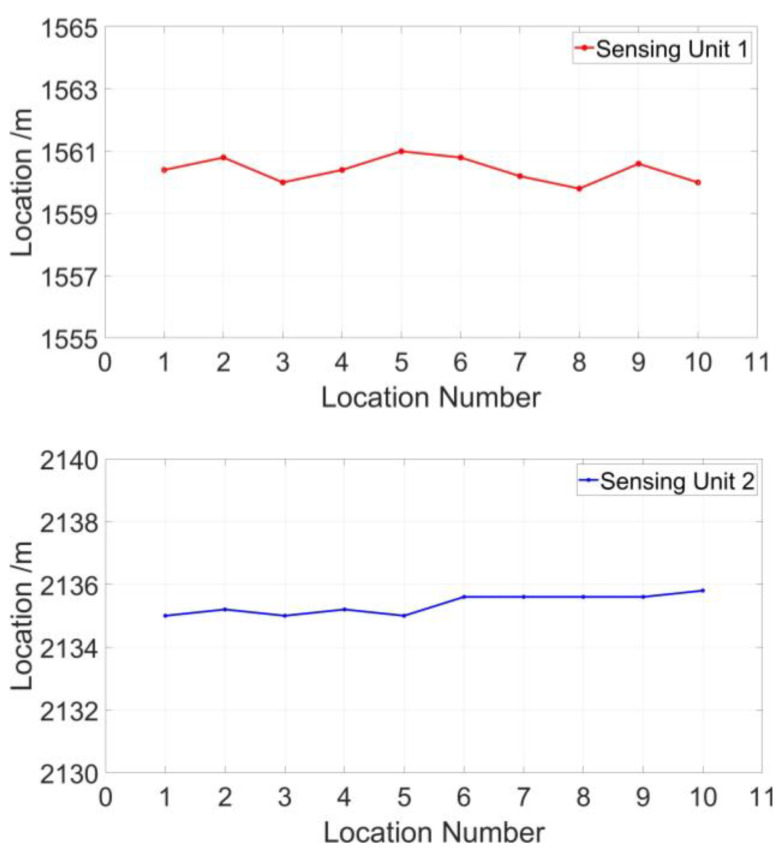
The location data of 10 locating tests for two sensing units.

As shown in Figure 7, the partial discharge signals were detected by sensing unit 1 and sensing unit 2, in which the length of the sensing optical fiber is 5400 m. In order to verify the location accuracy, we carried out 10 locating tests for the two sensing units. It is clear that there are two partial discharge locating units that have 10 locating peaks in each unit, and the locating peaks within each locating unit are very close to each other, as shown in Figure 8. As shown in Figure 9, the signal-to-noise ratio range of sensing unit 1 for 10 locating tests is from 23.2 to 33.9, while the signal-to-noise ratio range of sensing unit 2 for 10 locating tests is from 22.1 to 29.8. Therefore, the location performance of the sensing system is greatly improved after being processed by the time-domain variance locating algorithm. The measured location data of 10 locating tests for two sensing units are shown in Figure 10.

**Figure 11:**
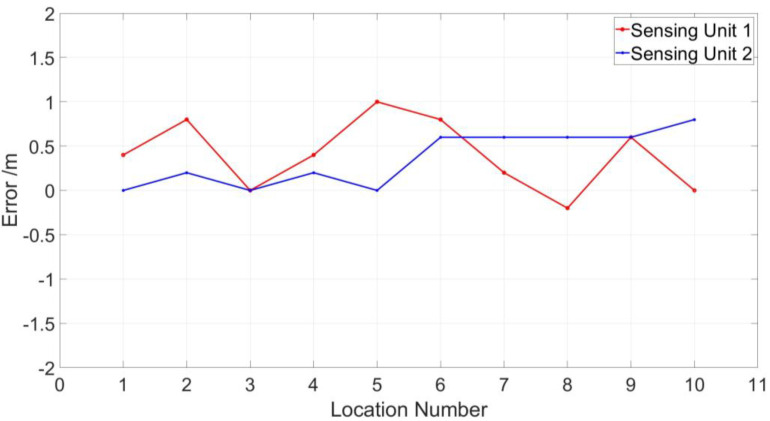
The errors of 10 locating tests for two sensing units.

As shown in Figure 10, the location range of sensing unit 1 for 10 locating tests is from 1559.8 to 1561.1, which has a 1.0-m maximum deviation from the actual position. The location range of sensing unit 1 for 10 locating tests is from 2135.0 to 2135.8, which has a 0.8-m maximum deviation from the actual position. The measured location errors and standard deviations of the two sensing units are listed in Table 1 and Figure 11.

**Figure 12:**
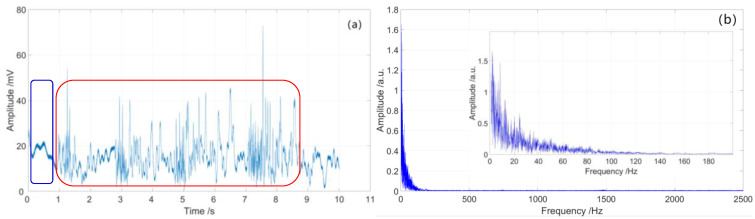
Partial discharge signal of sensing unit 1: (**a**) time-domain curve; (**b**) spectrum diagram.

**Figure 13:**
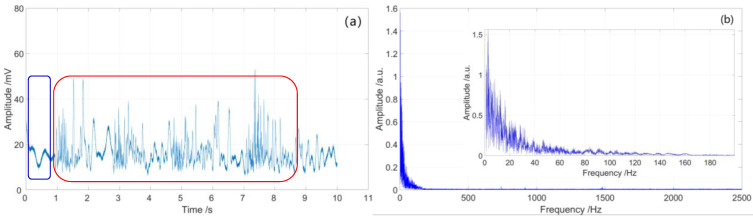
Partial discharge signal of sensing unit 2: (**a**) time domain curve; (**b**) spectrum diagram.

From Table 1 and Figure 11, we see that the maximum location error of sensing unit 1 is 1.0 m, and the maximum location error of sensing unit 2 is 0.8 m. The standard deviations of sensing unit 1 and sensing unit 2 are 0.3795 and 0.2939, respectively. Therefore, the partial discharge signals are extracted from raw Rayleigh backscattering signals according to location information, as shown in Figure 12a and Figure 13a.

From Figure 12a and Figure 13a, we see that the time-domain signal is mingled with system inherent noise and environment noise, which will reduce the quality of signal demodulation. The signal-to-noise ratios (SNR) of sensing unit 1 and sensing unit 2 are 4.5 and 2.8 dB, respectively. There are some disturbances in the time-domain vibration traces of the two sensing units, but the signals are fuzzy; note the red rectangles in Figure 12a and Figure 13a.

The spectrum range of the partial discharge signal is mainly below 200 Hz, as shown in Figure 12b and Figure 13b. There are some low-frequency noises with frequencies less than 5 Hz, which are caused by fluctuations in the system light source; note the blue rectangles in Figure 12a and Figure 13a.

**Figure 14:**
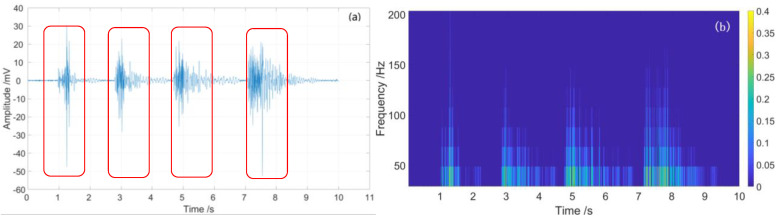
The demodulated partial discharge signal of sensing unit 1: (**a**) time domain curve; (**b**) spectrum diagram.

**Figure 15:**
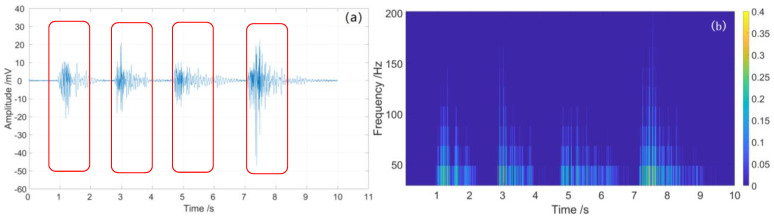
The demodulated partial discharge signal of sensing unit 2: (**a**) time-domain curve; (**b**) spectrum diagram.

For high-quality signal detection, the moving subtraction is used to demodulate partial discharge signal; furthermore, the high-pass filter is used to reduce the system low-frequency noise and environment noise. The demodulated time-domain curves and spectrum diagrams of the partial discharge signal are shown in Figure 14 and Figure 15.

**Figure 16:**
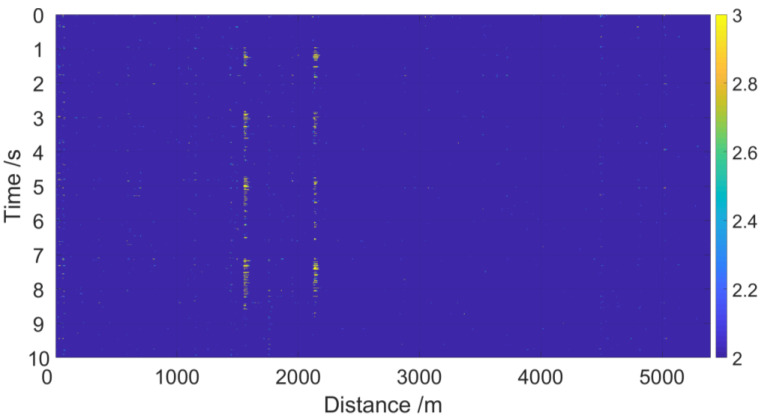
Energy waterfall diagram of four partial discharge signals at two sensing units.

**Figure 17:**
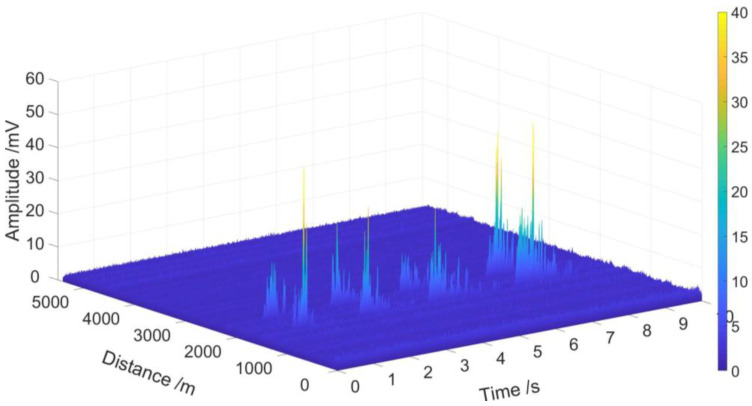
Three-dimensional energy diagram of four partial discharge signals at two sensing units.

From Figure 14a and Figure 15a, we see that four partial discharge signals were well detected by sensing unit 1 and sensing unit 2, respectively; note the red rectangles. The four partial discharge signals can also be clearly seen from the spectrum diagram, of which the frequency is mainly below 200 Hz, as shown in Figure 14b and Figure 15b. After demodulation, the signal-to-noise ratios (SNR) of sensing unit 1 and sensing unit 2 are greatly improved, which are 39.7 and 38.8, respectively, as shown in Table 2. The energy waterfall diagram and three-dimensional energy diagram show the four partial discharge signals clearly, as shown in Figure 16 and Figure 17.

## 5. Conclusions

In this paper, a distributed partial discharge sensing scheme based on optical fiber Rayleigh backscattering light interference was experimentally demonstrated. The proposed system can locate and detect partial discharge signals simultaneously. Compared with other partial discharge sensors, the distributed optical fiber partial discharge sensing system can not only detect multipoint discharge signals simultaneously but can also obtain their locations. We performed 10 locating tests for two sensing units. The experimental results show that the maximum location error of sensing unit 1 is 1.0 m, and the maximum location error of sensing unit 2 is 0.8 m. The standard deviations of sensing unit 1 and sensing unit 2 are 0.3795 and 0.2939, respectively. At the same time, the moving subtraction is used to demodulate partial discharge signals. Furthermore, the high-pass filter is used to reduce the system low-frequency noise and environment noise. It is clear that the proposed system can detect a partial discharge signal generated by metal needle sensitivity, and the detectable frequency range is 0–2.5 kHz. The signal-to-noise ratios (SNR) of sensing unit 1 and sensing unit 2 are greatly improved after demodulation, which are 39.7 and 38.8, respectively. This provides a new method for multipoint distributed optical fiber sensing location and detection of a long-distance electrical equipment partial discharge signal.

## Figures and Tables

**Table 1:** The data of 10 locating tests for two sensing units.

Number	Sensing Unit 1 (m)		Sensing Unit 2 (m)	
	Measured Location	Error	Measured Location	Error
1	1560.4	+0.4	2135.0	+0.0
2	1560.8	+0.8	2135.2	+0.2
3	1560.0	+0.0	2135.0	+0.0
4	1560.4	+0.4	2135.2	+0.2
5	1561.0	+1.0	2135.0	+0.0
6	1560.8	+0.8	2135.6	+0.6
7	1560.2	+0.2	2135.6	+0.6
8	1559.8	−0.2	2135.6	+0.6
9	1560.6	+0.6	2135.6	+0.6
10	1560.0	+0.0	2135.8	+0.8
Standard deviation	0.3795		0.2939	

**Table 2:** The signal-to-noise ratio before and after demodulation.

Status	Signal-to-Noise Ratio (SNR/dB)	
	Sensing Unit 1	Sensing Unit 2
Before demodulation	4.5	2.8
After demodulation	39.7	38.8
SNR improvement	35.2	36.0

## Data Availability

Not applicable.
